# Supplementary material for: Race and Outcomes to [177Lu]Lu-PSMA-617 in Advanced Prostate Cancer
Source: Cancers (Basel). 2025 Jun 12;17(12):1960. doi: 10.3390/cancers17121960 (PMC12191161; doi:10.3390/cancers17121960)
Supplement: Supplementary file 1 [file cancers-17-01960-s001.zip › cancers-3645101-supplementary.pdf]

SUPPLEMENTARY MATERIAL

Supplementary Table S1 – Baseline patient characteristics (White versus Non-White)

	**Total (N=654)**	**White (N=593)**	**Non-White (N=61)**	**P**
Age, median (range)	72 (46-95)	72 (46-95)	71 (51-91)	0.32
PSA at C1, ng/mL, median (range)	36 (0-8355)	32 (0-8355)	91 (0-1419)	0.01
Number of prior therapies (%), median (range)	4 (2,9)	4 (2, 9)	3 (2, 7)	0.93
2-4	477 (73)	431 (73)	46 (75)	
5	109 (17)	101 (17)	8 (13)	
≥6	68 (10)	61 (10)	7 (11)	
Sites of metastases (%)				
Bone	580 (89)	522 (88)	58 (95)	0.13
Lymph node	446 (68)	403 (68)	43 (70)	0.70
Liver	83 (13)	77 (13)	6 (10)	0.48
Lung	85 (13)	77 (13)	8 (13)	0.98
Visceral metastasis (%)	149 (23)	136 (23)	13 (21)	0.77

Supplementary Table S2 – PSA–PFS and Overall Survival, stratified by race (White versus Non-White)

	White	Non-White
**PSA – PFS**		
No. events / N	241 / 593	29 / 61
Median (95% CI), months	7.1 (6.9-7.8)	4.4 (3.8-8.8)
6-month PFS, % (95% CI)	59 (54-63)	49 (36-64)
Hazard ratio (95% CI)	ref	1.36 (0.90, 1.99)
p-value		0.12
Adjusted hazard ratio*, (95% CI)	ref	1.15 (0.78-1.70)
Adjusted p-value		0.50
**Overall Survival**		
No. events / N	184 / 593	23 / 61
12-month OS, % (95% CI)	61 (56-77)	54 (39-70)
Hazard ratio (95% CI)	ref	1.29 (0.83-1.99)
p-value		0.25
Adjusted hazard ratio* (95% CI)	ref	1.14 (0.73-1.78)
Adjusted p-value		0.57
Median follow-up time in alive patients, month (IQR)	9.2 (6.9, 12.0)	7.4 (4.2, 13.5)

* Adjusted hazard ratios were estimated after adjusting for age at treatment initiation, number of prior systemic therapies, sites of metastasis, and PSA levels at C1D1.

Supplementary Figure S1 – Kaplan-Meier curves for PFS (1A) and OS (1B) for White and Non-White patients

1A:

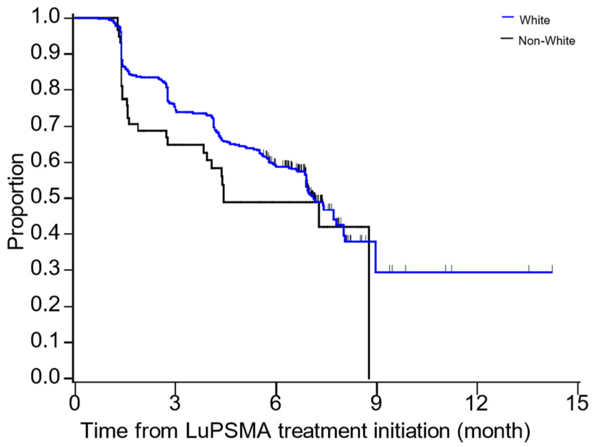

White	593	354	208	7	2	0
Non-White	61	31	16	0	0	0

1B:

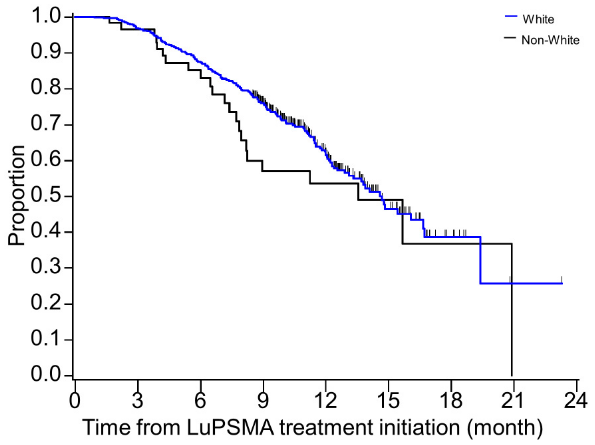

White	593	552	444	282	124	38	8	1	0
Non-White	61	56	38	19	16	4	1	0	0
